# Robo1/2 regulate follicle atresia through manipulating granulosa cell apoptosis in mice

**DOI:** 10.1038/srep09720

**Published:** 2015-05-19

**Authors:** Jiangchao Li, Yuxiang Ye, Renli Zhang, Lili Zhang, Xiwen Hu, Dong Han, Jiayuan Chen, Xiaodong He, Guang Wang, Xuesong Yang, Lijing Wang

**Affiliations:** 1 Institute of Vascular Biological Sciences, Guangdong Pharmaceutical University, Guangzhou 510006, China; 2 Key Laboratory for Regenerative Medicine of the Ministry of Education, Division of Histology & Embryology, Medical College, Jinan University, Guangzhou 510632, China; 3 Reproductive Medicine Center, Guangdong General Hospital, Guangzhou 515006, China; 4 Southern Medical University, Guangzhou 510515, China

## Abstract

Secreted Slit proteins and their Roundabout (Robo) receptors act as a repulsive cue to preventaxons from migrating to inappropriate locations during the development of the nervous system. Slit/Robo has also been implicated in reproductive system development, but the molecular mechanism of the Slit/Robo pathway in the reproductive system remains poorly understood. Using a transgenic mouse model, we investigated the function of the Slit/Robo pathway on ovarian follicle development and atresia. We first demonstrated that more offspring were born to mice with a partial knockout of the *Robo1/2* genes in mice. We next showed that Robo1 and Robo2 are strongly expressed in ovarian granulosacells. Apoptosis in granulosa cells was reduced when Robo1/2 were partially knocked out, and this observation was further verified by *in vitro* Robo1/2 knockout experiments in mouse and human granulosa cells. We also found that ovarian angiogenesis wasenhanced by a partial lack of Robo1/2 genes. In summary, our data suggest that the Slit/Robo pathway can impact follicle development and atresia by influencinggranulosa cell apoptosis.

The ovariesare a pair of ductless female reproductive organsin which female germ cells are generated. The ovarian follicle is the fundamental functional unit of the ovary andis composed of oocytes, granulosacells, and thecacells. Each primordial folliclehas the potential to undergo folliculogenesis and to develop into a primaryfollicle, a secondary follicle, and finally intoa mature folliclethat can ovulate, or degenerate in a manner similar to most other follicles that are not selected for maturation^1^. At birth, the mammalian ovary contains numerous of primordial follicles, and these begin to undergo folliculogenesis during puberty and will eventually be ovulated or will degrade in a process known as atresia. The decision as to whether a follicle will develop or ovulate or undergo atresia is regulated by a variety of factors^2^,^3^,^4^.

Slit was initially identified in *Drosophila* as a secreted protein thatmodulates the growth of glia cells at the midline during the development of the central nervous system^5^. The receptor of Slit, Roundabout (Robo), is a transmembrane protein that is predominately expressed on the axon growth cones in the central nervous system^6^. Slit/Robo signaling is fundamental in the repulsion of axons away from the midline in both invertebrates and vertebrates and thus plays a key a role in axon guidance at the midline of the central nervous system^7^,^8^. Slit/Robo signaling is not restricted to the development of the nervous system, and it has also been shown to functionin the development of the lung, kidney, and mammary gland. In addition to its physiological functions during embryonic development, Slit/Robo signalinghas also been implicated in a variety of pathological conditions such as cancer and inflammation^9^,^10^. The mammalian Slit family consists of Slit1, Slit2, and Slit3. All three proteinsareexpressed in the ventral neural tube during neurulation, but the expression patterns are not exactly the same. Slit-1 is primarily expressed in the nervous system, butSlit2 and Slit3 are present in tissues other than the nervous system^11^,^13^. The Robo receptor family consists of Robo1, Robo2, Robo3, and Robo4.

Dickenson et al. reported that Slit/Robo signaling also plays an important role in the reproductive system^13^. The authorsused RT-PCR to showthat Slit2, Slit3, Robo1, Robo2, and Robo4 were expressed in sheep ovaries during gestation, and they demonstrated that the expressionlevels of Robo2 and Robo4 were elevated during the early stages of follicle formation and remained high throughoutfollicle maturation. More interestingly, Robo1 was found to be expressedin the pre-granulosacells, whereas Robo2, Robo4, and Slit2 were expressed in growing oocytesof the developing primordial follicle^14^. Steroid hormones modulate Slit/Robo, which subsequently regulates reproductive functions in the ovary and endometrium^13^. However, the precise role of Slit/Robo in the regulation of physiological and pathophysiological ovarian functions remains poorly understood.

In this study, we aimed to assess the role of Slit/Robo signaling in ovarian follicle development and atresiausing Robo1/2 transgenic mice. Based on the observed effect that Robo1/2^+/−^ knockout affected fertilityin mice, we carefully analyzed follicular development and atresia in Robo1/2-deficient mice and found a correlation with granulosacell apoptosis and ovarian angiogenesis. Our experimental data suggest that Slit/Robo signaling might be involved in the regulation of folliculogenesis by regulating apoptosis in granulosa cells.

## Methods

### Transgenic mice

Robo1^+/−^ and Robo2^+/−^ double knockout transgenic mice(Robo1/2^+/−^, Strain Name: STOCK Robo1tm1Matl Robo2tm1Mrt/MatlMmmh) were purchased from the MMRRC (Mutant Mouse Regional ResourceCenters, catalog, 030747-MU, https://www.mmrrc.org, San Diego, USA). The mice were genotyped by PCR. TheRobo1 primers wereF1: TGG CAC GAA GGT ATA TGT GC; F2: GAA GGA CTG GTG GTT TTG AG; and F3: CCTCCGCAAACTCCT ATTTC. TheRobo2 primers wereF4: AAG TGC AAC GTC TCT GAA GTC CC; F5: GGC GGA ATT CTT AAT TAA GGC GCG; and F6: TTC TTT AGA AGG CAC AAC AAT CTC AGA G. For details regarding the genotyping protocols, please refer to the websitehttps://www.mmrrc.org/catalog/sds.php?mmrrc_id=30747. Male wild-type mice were mated with Robo1/2^+/−^ female mice. Robo1^−/−^ and Robo2^−/−^ mice are embryonic lethal. The mice were housed at 25°C ± 1°C, 50% ± 10% humidity, and a 12 h: 12 hlight: dark cyclein a specific pathogen-free animal facility at the Animal Center, Guangdong Pharmaceutical University, Guangzhou, China. All methods were carried out in accordance with the approved guidelines and all animal experimental protocols were approved by the animal experimental ethics committee of Guangdong Pharmaceutical University.

### Histology

Transgenic mouse ovaries were randomly collected from wild-type and Robo1/2+/− groups. Following immersion in 4% paraformaldehyde at 4°C for 12 h, the ovaries were embedded in paraffin wax. For hematoxylin and eosin staining (H&E) and immunohistochemistry(IHC), the serial sections (5 μm) from each ovary were aligned in order on glass microscope slides. The follicles of each ovary from the different groups were then categorized and counted.

IHC and immunofluorescence (IF) were performed as described on the website of Cell Signaling Technology, Inc. (CST, MA, USA). In brief, the slides were pre-treated with EDTA solution (pH = 9.0) for antigen retrieval and then incubated with the primary antibody at 4°C overnight. The primary antibodies included Ki67 (1:100 dilution, Abcam, USA), CD34 (1:100, Boster, China), Robo1 (1:100, SantaCruz, USA), Robo2 (1:100, Santa Cruz, USA), and FSHR (1:200, Santa Cruz, USA). Secondaryantibody detection was with the Universal LSAB Kits (Dako, Denmark) using horseradish peroxidase (HRP). Rabbit and mouse antibodies with HRP were purchased from Dako. For IF staining, the slides were incubated with monoclonal CD31 (1:100, SantaCruz, USA) or monoclonal SMA (1:100, Boster, China) antibodies followed by a specific secondary antibody mixture coupled with Alexa Fluor 555 or Alexa Fluor 488 anti-rabbit IgGand then counterstained with DAPI (4′-6-diamidino-2-phenylin-dole, 10 ng/mL, Invitrogen, USA) for 15 min at room temperature.

### Follicle categorizationandcounting

The follicles were categorized as primordial, primary, secondary, mature, oratretic. Follicles were classified as primordial if they contained an oocyte surrounded by a single layer of flattened follicular cells. When the flattened cells of the follicles became squamous or cuboidal—which are known as granulosacells—these follicles were classified as primary follicles. Secondary follicles were identified by the presence of visible follicular antrum, and in follicles with a markedly enlarged antrum, the cumulus oophorus diminishes there by leaving the oocyte surrounded by a 2–3 layergranulosacells. And oocytefloats freely inside the follicle. After this stage, the follicle bulges outward from the ovary and is classified as a mature follicle. Typical interstitial glands and follicles with a shrunken oocyte or with granulosacellsthat had begun to disaggregate were categorized as atreticfollicles. The total number of follicles per ovary was determined by taking the average of the counts from three sections (five sections apart) cut along the long axis of the whole ovary.

### Detection of apoptosis

The extent of cell death in the ovary was established by TUNEL analysis using an In Situ Cell Death Detection Kit (Roche, USA). The staining was performed according to the protocol provided by the manufacturer, which we adapted for labeling tissue slides. TUNEL-positive cells were counted using Image Analysis Software (Olympus, Japan). At least three ovaries were assayed per groupinindependent experiments.

### Human granulosacell collection and culture

The granulosacells were obtained from the follicular fluidunder microscope with graduated pipette, using 1× PBS to completely remove anyblood cells. The cells were centrifuged at 2000 g, 10 min, the supernatant was discarded, and hyaluronidase (80 U/ml, SAGE IVF Inc., CT, USA) was addedfordigesting protein and obtained the single granulosa cells with 400 mesh filter. Then the DMEM medium with 20% FBS and penicillin-streptomycinwas added to culture the cells, and the cells were used to inoculate culture dishes. The methods of obtained follicular fluid were carried out in accordance with the approved guidelines (In vitro fertilization), the discarded the follicular fluid were collected from women after performed in-vitro fertilization in the Reproductive Medicine Center, Guangdong General Hospital (Guangzhou, China). Informed consent was obtained from all subjects and this study was approved by the ethics committee of Guangdong General Hospital.

### Mousegranulosa cell collection and primary culture

For mouse granulosacells, the mice received an intraperitoneal injection of 10 U pregnant mare serum gonadotropin (PMSG). After 48 hours, 10 U human chorionic gonadotropin (hCG) was injected. Sixteen hours later, the mice were sacrificed. Several bilateral ovarian follicles were removed from the abdominal cavity surface and washed with sterile 1× PBS. A syringe needle was used to puncturethe mature follicles to release the granulosacells. The cells were cultured with high-glucose DMEM that contained 10% fetal bovine serum at 37°C in 5% CO_2_ in a humidified incubator.

### siRNA interference

The siRNA and control (mock siRNA) were transfected into a series of cells using the lipofectamine 2000 reagent (Invitrogen, USA) according to the manufacturer's instructions after they had been cultured for 48 hours. Human Robo1/Robo2 siRNA and control siRNA were purchased from Riobio, Inc. (Guangzhou, China). Human Robo1-siRNA: GGATGTATTTGCAACAAGATT; HumanRobo2-siRNA: CACCATTGAGTGGTACAAAGATG.

### Flow cytometry

Ovarian tissuesofRobo1/2^+/−^ knockout mice and wild-type mice were minced into 1 mm^3^ pieces to obtain granulosacells and then 0.25% trypsin and 0.02% EDTA were added to digest the proteinholding the granulosa cells. The samples were centrifuged at 800 rpm for 5 min. To investigate if Robo1 and Robo2 affect granulosa cell apoptosis, the Flow cytometry was performed according to the Annexin V/PI apoptosis assay kit protocol (Catalog: AP101-30, Multiscience Company, USA).

### Image analysis

The paraffin-embedded tissues of mouse ovaries were sectioned into 5 μm slices using a rotary microtome (LeicaRM2126RT). H&E staining, IHC, or IF staining were performed on the sections. All experiments were photographed with an Olympus IX51epi-fluorescent microscope (at 200× and 400× magnification) and analyzed using the CW4000 FISH Olympus software. For image analysis and scoring, Image-Pro Plus6.0 (IPP6.0) professional imaging software was used. The positive-staining areas in the images were measured within an integrated optical density (IOD), and these values were used to analyze and compare all protein expression-positive cellsand TUNEL-positive cells.

### In vitro fertilization experiment

The mice were injected with 10 U PMSG. After 48 h, 10 U hCG was administered. Following superovulation, eggs were collected from the ampulla of the uterine tube and co-cultured with wild-type mouse sperm. After 6–8 hours, the fertilized eggs were identified and evaluated. All sperm were obtained from the same male mouse.

### Western blot

The cultured cells and tissues were harvested and lysed inice-cold cell lysis buffer that included 50 mM Tris-HCl (pH 7.4), 150 mM NaCl, 10% glycerol, 1% Triton X-100, 2 mM EDTA, 2 mM EGTA, 40 mM β-glycerophosphate, 50 mM sodium fluoride, 10 mg/mL leupeptin, 10 mg/m Laprotinin, 1 m Mpepstatin A, and 1 mM phenylmethylsulphonyl fluoride. A total of 30 mg protein was added to each lane and separated by 8% SDS-PAGE. The bands were transferred to a PVDF membrane (Millipore Corporation, USA) that was then blocked with 5% nonfat dry milk for 1 hour at room temperature and incubated with anti-Robo1 (1:500, Santa Cruz), anti-Robo2 (1:500, Santa Cruz), and GAPDH(1:5,000, Santa Cruz) overnight at 4°C. The samples were further incubated with HRP conjugated anti-rabbit secondary antibodies (1:10,000, Santa Cruz), and the signal was detected by an ECLkit(Millipore, USA) and developed on X-ray film. GAPDH antibody was used as the internal control.

### Hormone test

Serum hormone levels were determined with achemiluminescence immunoassay method that was carried outin the Clinical Laboratory of the Reproductive Medicine Center of Guangdong GeneralHospital.

### Chemotherapy

Eight-week-old female mice (wild-type mice, n = 10; Robo1/2^+/−^ mice, n = 10) were administered an intraperitoneal injection of cyclophosphamide (Cy) (75 mg·kg^−1^·day^−1^ for 4 weeks) or PBS. Mice weresacrificed one week after treated by Cy. Two weeks after the final treatment, the female mice were mated with wild-type mice for oneweek. Mating was confirmed by the formation of the fertilization plug (within one week).

### Statistical Analysis

The results are presented as the mean value (mean ± standard deviation (SD)). Data analyses and graphing were performed using the GraphPad Prism 5 software (GraphPad Software, CA, USA). The images of IHC and IF were analyzed with IPP6.0 software. Pearson's Chi-square test was used to compare theIFresults. The real-time PCR data were analyzed using Student's *t*-test. A *p*-value less than 0.05 was considered significant.

## Results

### Robo1/2^+/−^ knockout increased fertility in mice

**Figure 1:**
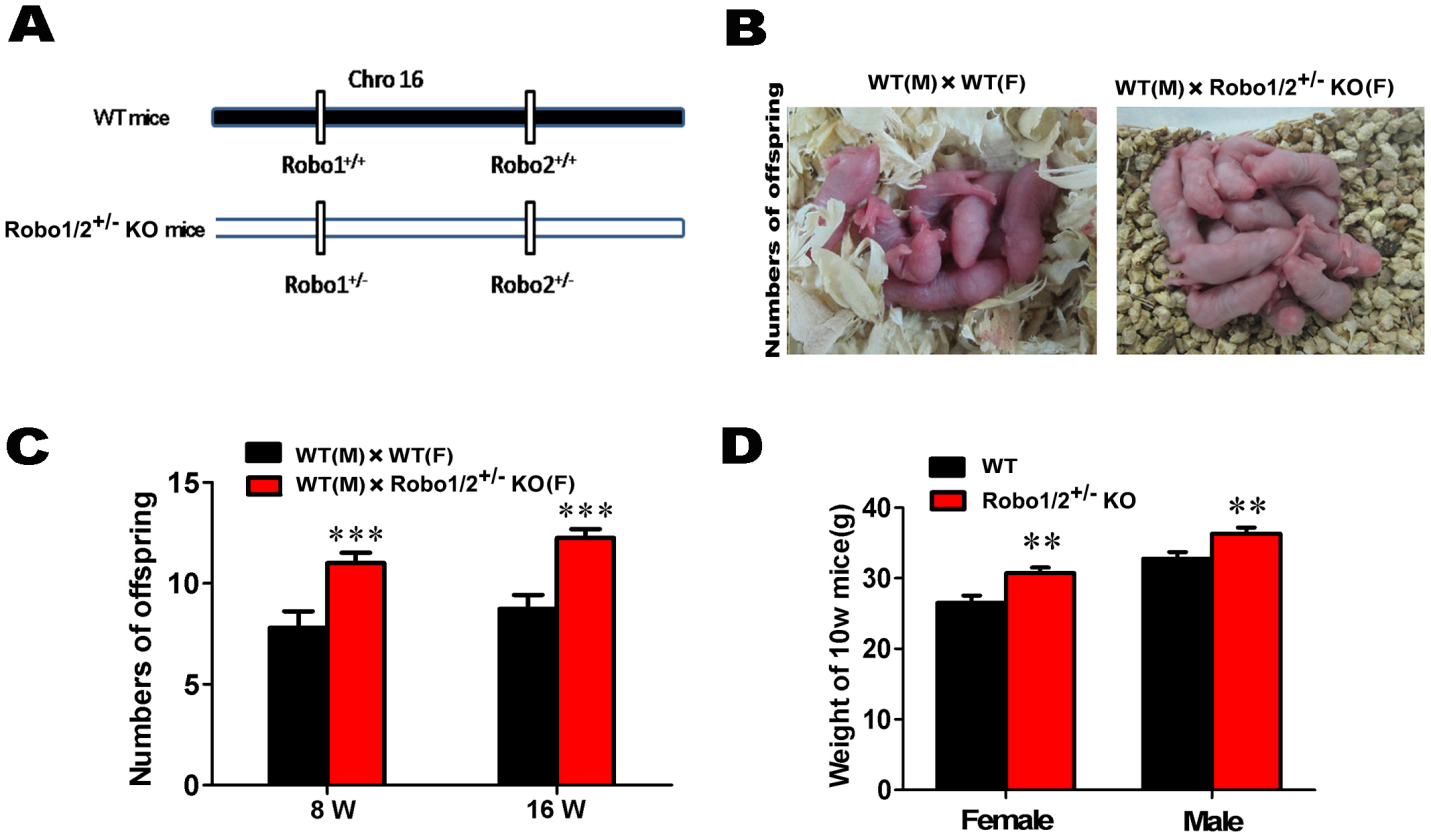
The general phenotype of the Robo1/2 knockout transgenic mice. (A): The chart shows the Robo1 and Robo2 mutation sites in chromosome 16. (B): The photographs show the typical number of offspring in male WTcrossbreed with Robo1/2^+/−^ knockoutmice(female). (C): The chart shows the comparison of offspring number between WT(male) × WT(female)mice withWT(male) × Robo1/2^+/−^ knockout mice(female) (WT 8-week-old n = 10, Robo1/2 8-week-old n = 13; WT 16-week-old n = 19, Robo1/2 16-week-old n = 27). (D): The chart shows the weight comparison between the 10-week-old wild-type and Robo1/2^+/−^ knockout mice (WT female n = 7, Robo1/2 female n = 12, WT male n = 15, Robo1/2 male n = 19). **p < 0.05 and ***p < 0.001 indicate significant differences between the wild-type and Robo1/2^+/−^ knockout groups. Abbreviations: Chro, chromosome; WT, wild-type; Robo1/2^+/−^, double Robo1/2^+/−^ knockout.

Dickinson et al. reported that Slit/Robo signaling was active during fetal ovary development and suggested that it might function in autocrineorparacrine interactions^13^,^14^,^15^. Because of the embryonic lethality when Robo1/2 areknocked out [19–20]. Knocked out Robo1 and Robo2 both located on chromosome 16 as shown in Fig. 1A. Interestingly we found that the number of offspring in the Robo1/2^+/−^ knockout mice was greater than the wild-type mice (Fig. 1B). To exclude any differences at different age, we analyzed the number of offspring atweek8 and week 16 and observed the same increasein offspring in theRobo1/2^+/−^ knockout mice at both time points (Fig. 1D). In addition, the weights of both female and male mice at 10 weeks of age were enhanced in Robo1/2^+/−^ knockout mice compared with the wild-type controls (Fig. 1D), but the mechanism behind this weight gain is unknown.

To further confirm this observation, Cy, a chemotherapeutic and immunosuppressive agent for the treatment of some neoplastic and autoimmune diseases (details in Materials and Methods), was administered to the wild-type and Robo1/2^+/−^ knockout mice at 8 weeks of age because Cy has been reported to disturb follicle growth and toresult in premature menopause and sterility athigh doses^16^,^17^,^18^. The data shown in Supplementary figure 1A indicate that Robo1/2 knockout mice were able to rescue the Cy-induced reduction in offspring number. In the subsequent TUNEL assay with mice treated with Cy for 4 weeks, we found that theRobo1/2^+/−^ knockout could prevent the apoptosis that is induced by Cy treatment (Supplementary fig. 1C). We also calculated the number of various stages of follicles following the injection of Cy (Supplementary fig. 1B).

### Robo1/2^+/−^ knockout had a small impact on ovarian hormone secretion and gamete viability

To explore the potential mechanism behind the enhancement of fertility that was induced by Robo1/2^+/−^ knockout, we first assessed gameteviability by measuring the rates of ovum maturation and fertilizationand the rates of zygote cleavage and degradation (Supplementary fig. 2). All determinations were performed in 4-week-old (Supplementary fig. 2A–B) and 10-week-old (Supplementary fig. 2E–F) mice, and we found no significant differences between the wild-type and Robo1/2^+/−^ knockout mice at either age.

**Figure 2:**
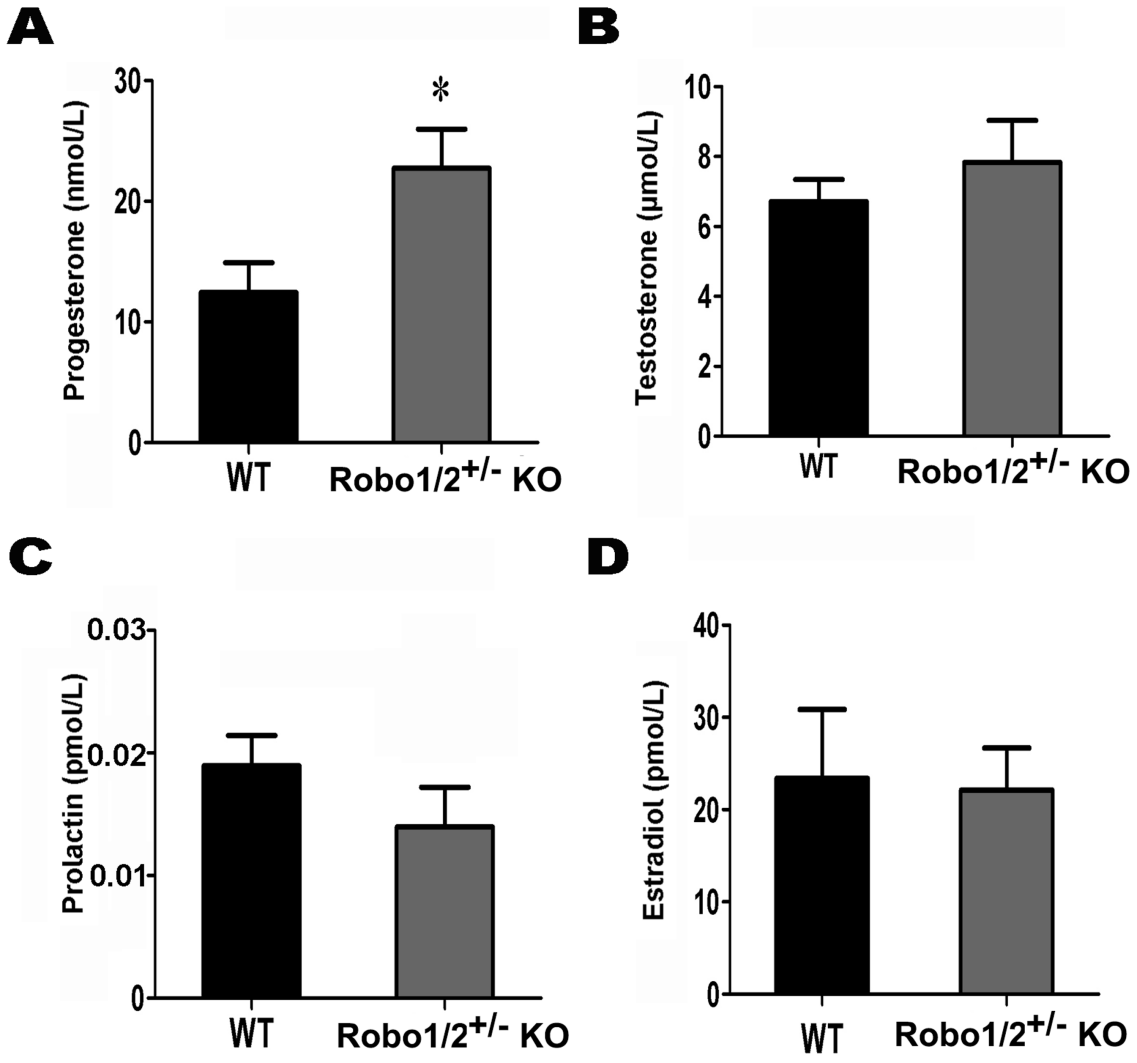
The hormone determination in wild-type and Robo1/2^+/−^ knockout mice. The progesterone, testosterone, prolactin, and estradiol levels in wild-type (WT, n = 8) and Robo1/2^+/−^ knockout (ROBO1/2^+/−^ knockout mice, n = 9) mouse blood were determined. (A): The blood progesterone levels in wild-type and Robo1/2^+/−^ knockout mice. (B): The blood testosterone levels in wild-type and Robo1/2^+/−^ knockout mice. (C): The blood prolactin levels in wild-type and Robo1/2^+/−^ knockout mice. (D): The blood estradiol levels in wild-type and Robo1/2^+/−^ knockout mice. *p < 0.05 indicates a significant difference between the wild-type and Robo1/2^+/−^ knockout groups.

Female fertility relies on the regulation of both pituitary and ovarian sex hormones. We measured the levels of prolactin, progesterone, estradiol, and testosterone in the blood of 10-week-old wild-type and Robo1/2^+/−^ knockout mice (Fig. 2). Unfortunately, we failed to successfully detect FSH and luteinizing hormone(LH), and this was most likely because of the difficulty incollecting sufficient amounts of mouse blood or the insensitivity for both FSH and LH. The results did indicate, however, that Robo1/2^+/−^ knockout led to an increase in progesterone secretion (Fig. 2A) but to no significant changes in the secretion of pituitary prolactin orovarian estradiol ortestosterone (Fig. 2B–D). Sothe findings suggest that the changes in fertility induced by Robo1/2^+/−^ knockout are probably not only the result of interference with hormone secretion.

### Robo1/2^+/−^ knockout accelerated ovarian follicle maturation

**Figure 3:**
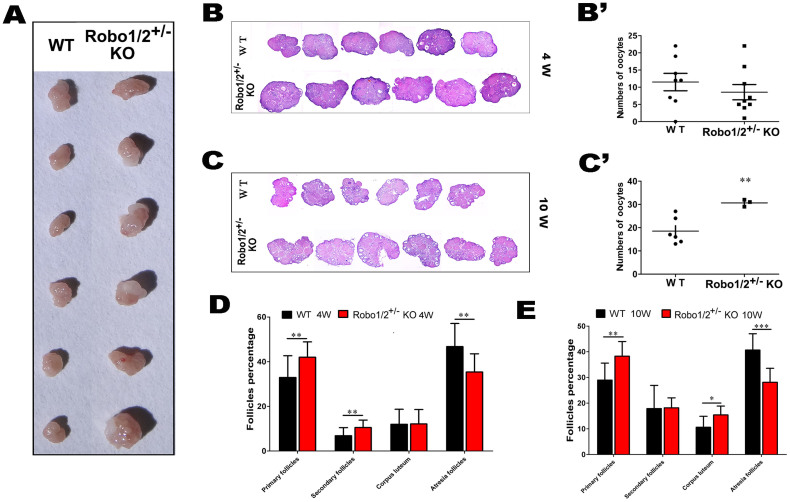
The Robo1/2^+/−^ knockout ovary promoted follicle maturation. (A): The two groups of ovaries from the 10-week-old wild-typeand Robo1/2^+/−^ knockoutmice. (B): H&E staining of ovarian vertical sections from 4-week-old wild-type and Robo1/2^+/−^ knockout mice. (C): H&E staining of ovarian vertical sections from 10-week-old wild-type and Robo1/2^+/−^ knockout mice. (B′–C′): The diagrams show that the number of oocyte induced by hyperstimulation are not apparently changed in the 4-week-old (B′, WT n = 8, Robo1/2 n = 9) but increase in 10-week-old (C′, WT n = 6, Robo1/2 n = 3) Robo1/2^+/−^ knockout mice, respectively. (D–E): Bar chart showing the changes in 4-week (D, WT n = 17, Robo1/2 n = 15) and 10-week (E, WT n = 8, Robo1/2 n = 10) ovarian follicle number in terms of follicle stage, including primary follicles, secondary follicles, and corpus lutea. The total follicle number was also counted. ***p < 0.001 indicates a significant difference between the wild-type and Robo1/2^+/−^ knockout groups. Abbreviations: WT, wild-type; ROBO1/2^+/−^ mice, double Robo1/2^+/−^ knockout. Scale bars = 200 μm in (A) and 500 μm in (B and C).

One possibleway to enhance fertility is to alter ovarian follicle development. Therefore, we compared the ovarian follicle development in the wild-type and Robo1/2+/− knockout mice (Fig. 3). First, we foundthat the ovary weight dramatically increased in the Robo1/2+/− knockout mice compared with the wide-type mice (data not shown), and Figure 3A showssix examples of ovaries from the wild-type and Robo1/2+/− knockout mice. The differences in ovary size could also be observed in the H&E staining of vertical sections of the ovaries from4-week-old (Fig. 3B) and 10-week-old (Fig. 3C) mice. To observe the differences in the number of oocytes, ovulation was induced by hyperstimulation. The results showed that there was no increase in oocyte number in wide-type (Fig. 3B′) but increase in10-week-oldRobo1/2^+/−^ knockout mice (Fig. 3C′). However, the oocyte number was significantly increased in the 10-week-oldRobo1/2+/− knockout mice compared with the control mice (Fig. 3C′). This phenotype was the impetusto check the ovarian follicle development at different stages (Fig. 3D–E). We try to explain that phenotype (more litter in Robot1/2+/− mice) using primordial focllicles, but it is difficult to evaluation in cell morphology, and the number is not apparently different in wild-type mice compared to Robo1/2+/− knockout mice (data not shown). We determined that there were more primary and secondary follicles in both the 4-week-old and 10-week-oldRobo1/2+/− knockout mouse ovaries compared with the wild-type mouse ovaries (Fig. 3D–E), although the total number of corporalutea and the total number of folliclesin the Robo1/2+/− knockout ovaries only increased in the 10-week-old mice, not the 4-week-old mice (Fig. 3D–E). This finding implies that the level of Robo1/2+/− knockoutpromotesfollicle maturation and ovulation; thus, Slit/Robo signaling undernormal physiological conditions restrains follicle maturation or modulates follicle atresia.

### Robo1 and Robo2 are robustly expressed in ovarian granulosa cells in wild-type mice

**Figure 4:**
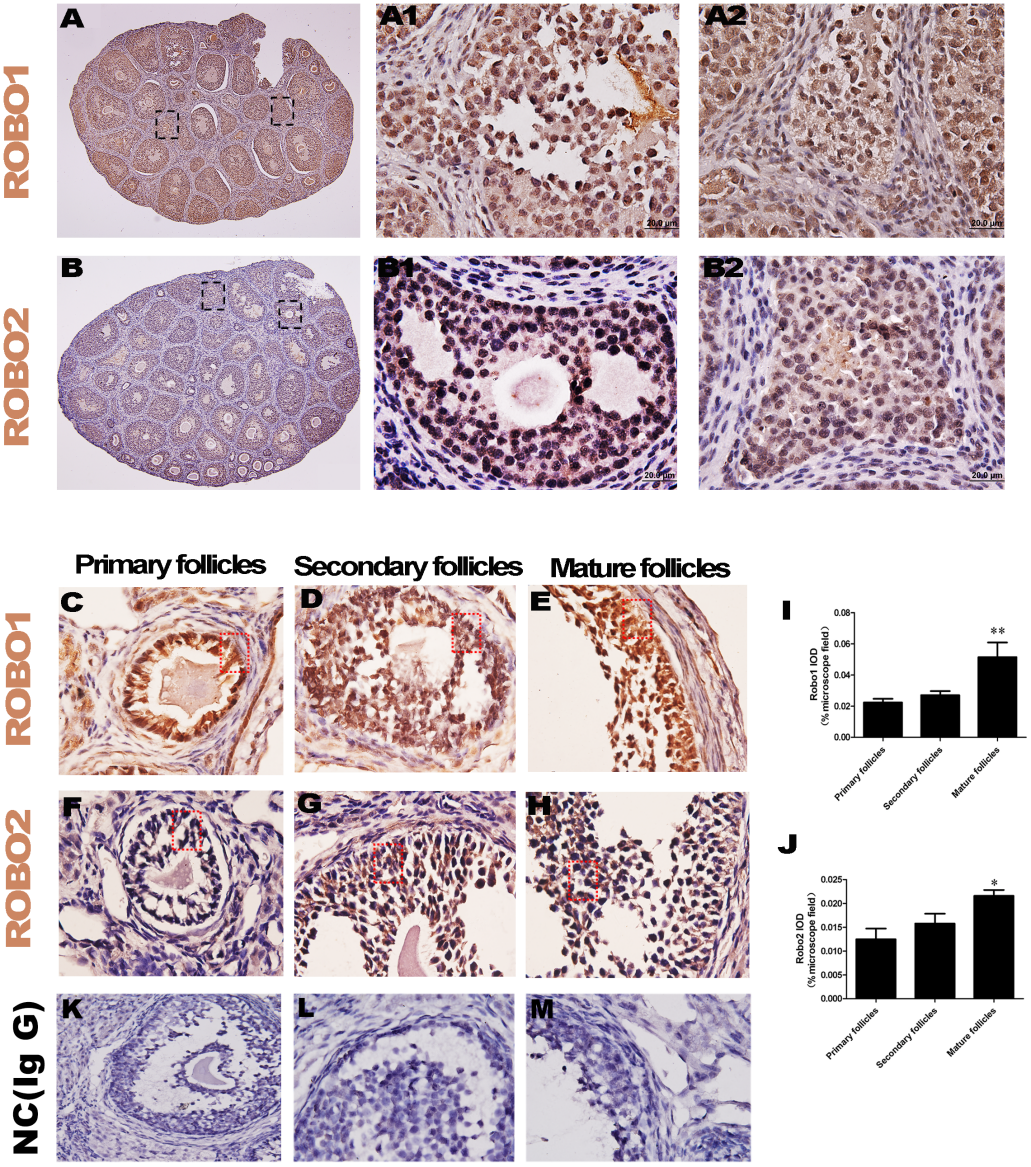
Robo1 and Robo2 are primarilyexpressed in ovarian granulosa cells. (A–B): The immunocytochemistry against Robo1 (A) and Robo2 (B) was performed on the vertical sections of the ovaries. (A1–A2): The high-magnification images of Robo1 expression as indicated by the dotted squares in (A). (B1–B2): The high-magnification images ofRobo2 expression as indicated by the dotted squares in (B). (C–E): The high-magnification images ofRobo1 expression in a primary follicle (C), a secondary follicle, (D) and a mature follicle (E). (F–H): The high-magnification images ofRobo2 expression in a primary follicle (F), a secondary follicle, (G) and a mature follicle (H). (I): The bar chart showing the comparison of integral optical density (IOD) for Robo1 expression in primary (n = 10), secondary (n = 5), and mature (n = 5) follicles. (J): The bar chart showing the comparison of the IOD for Robo2 expression in primary (n = 8), secondary (n = 5), and mature (n = 5) follicles. *p < 0.05 and **p < 0.01 indicate significant differences between the WT and Robo1/2^+/−^ knockout groups. Scale bars = 200 μm in (A–B) and 20 μm in (A1–A2), (B1–B2), and (C–H). Negative control group: mouse IgG(K, L, M).

In theRobo1/2^+/−^ knockout mice, we demonstrated that the lack of Robo1/2 increased the number of corporalutea, and this suggests that ovulation was promoted by the down-regulation of Robo1/2 expression. In wild-type mice, the IHC analysis of Robo1 and Robo2 showed that both receptors are expressed in the ovary (Fig. 4A–B), especially in ovarian granulosa cells (Fig. 4A–A2, B1–B2). Figures 4C–E clearly showthat Robo1 was expressed in the granulosa cells of the primary follicle (Fig. 4C), the granulosa cells in the secondary follicle (Fig. 4D), and the granulosa cells in the mature follicle (Fig. 4E). Figures 4F–H clearly show that Robo2 was also expressed in the granulosa cells of the primary follicle (Fig. 4F), the granulosa cells of the secondary follicle (Fig. 4G), and the granulosa cells ofthe mature follicle (Fig. 4H). To quantity the expression levels of Robo1 and Robo2 in the different stagefollicles, the IOD was calculated from the regions indicated by red dotted squares in Figures 4C–H. The results indicated that Robo1 expression (Fig. 4I) and Robo2 expression (Fig. 4J) appeared to be increasing as the follicle matured. These findings suggest that there is endogenous Robo1 and Robo2 expression in granulosa cells and that these receptors play a crucial role in the maintenance of follicle maturation and atresia.

### Robo1/2^+/−^ knockout reduced apoptosis in ovarian granulosa cells

**Figure 5:**
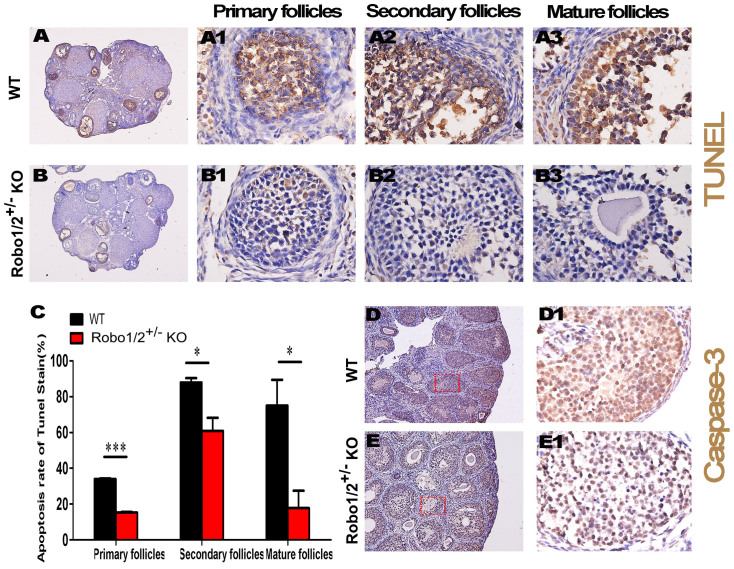
Apoptosis in granulosa cells is reduced in Robo1/2^+/−^ knockout mice. (A–B): The detection of apoptosis by the TUNEL assay was performed on the vertical sections of ovaries obtained from wild-type (A) and Robo1/2^+/−^ knockout (B) mice. (A1–A3): The high-magnification images from a wild-type ovary (A) showing TUNEL staining in a primary follicle (A1), a secondary follicle (A2), and a mature follicle (A3). (B1–B3): The high-magnification images from a Robo1/2^+/−^ knockout ovary (B) showing TUNEL staining in a primary follicle (B1), a secondary follicle (B2), and a mature follicle (B3). (C): The bar chart showing the percentage of TUNEL-positive apoptotic granulosa cells in primary (n = 3), secondary (n = 3), and mature (n = 3) follicles from the wild-type and Robo1/2^+/−^ knockout mouse ovaries. (D–E): Immunochemistry against caspase-3 was performed on vertical sections of the wild-type (D) and Robo1/2^+/−^ knockout (E) ovaries. (D1–E1): The high-magnification images from the sites indicated by red squares in (D and E), respectively. *p < 0.05 and ***p < 0.001 indicate significant differences between the wild-type and Robo1/2^+/−^ knockout groups. Abbreviations: WT, wild-type; ROBO1/2^+/−^ mice, double Robo1/2^+/−^ knockout. Scale bars = 500 μm in (A–B) and 20 μm in (A1–A3) and (B1–B3).

We measured changes in ovarian follicle development following the partial knockdownof Robo1/2. Apoptosis in the granulosa cells was closely associated with the dominant follicle selection and follicular atresia, thus we measured cell apoptosis in the Robo1/2^+/−^ knockout ovaries using a TUNEL assay (Fig. 5). Apoptosis in the ovarian granulosa cells of the Robo1/2^+/−^ knockout mice was dramatically reduced compared with the wild-type mice (Fig. 5A–0B). The reduction in apoptosis induced by the partial knockdown of Robo1/2 was observed in follicles at various stages of development, including primary (Fig. 5A1–B1), secondary (Fig. 5A2–B2), and mature follicles (Fig. 5A3–B3). In addition, IHC against caspase-3, another apoptosis marker, also indicated that a Robo1/2^+/−^ knockout could reduce cell apoptosis compared with the wild-type mice (Fig. 5D–E, D1–E1).

**Figure 6:**
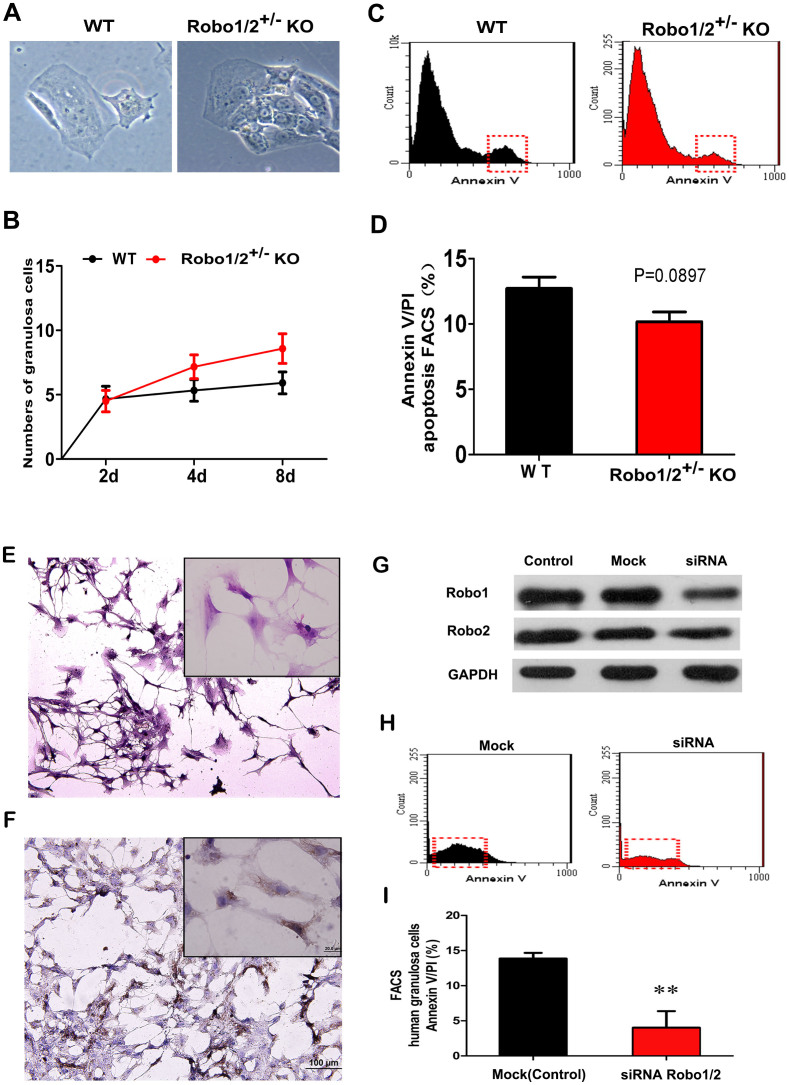
The reduced apoptosis in granulosa cells following Robo1/2^+/−^ knockout was verified in vitro. (A–D): Mouse granulosa cells were used in these experiments. (A): The mouse ovaries were isolated and dissociated into single granulosa cells from wild-type mice and Robo1/2^+/−^ knockout mice. (B): The graph showing the total numbers of granulosa cells isolated from the wild-type (n = 12) and Robo1/2^+/−^ knockout (n = 12) mice on culture days 2, 4, and 8. (C): The flow cytometry assay showing the population ratio of granulosa cells in different cell cycle stages. (D): The bar chart showing the percentages of apoptotic cells among wild-type (n = 3) and Robo1/2^+/−^ knockout (n = 3) mouse granulosa cells. (E–I): Human granulosa cells were used in these experiments. (E): H&E staining was performed in the primary culture of human granulosa cells, and a high magnification image is shown in the top right corner. (F): FSHR immunochemistry was performed in the primary culture of the human granulosa cells, and a high magnification image is shown in the top right corner. (G): Western blot showing that Robo1 and Robo2 were partially knocked down by Robo1-siRNA and Robo2-siRNA, respectively, compared with the control or mock siRNA (negative control). (H): The flow cytometry data showing the apoptotic cell population in the mock and Robo1/2^+/−^ knockout granulosa cells. (I): The bar chart showing the comparison of the apoptotic cell populations in the mock and Robo1/2^+/−^ knockout granulosa cells (Repeated at least 4 times each groups). Abbreviations: WT, wild-type; ROBO1/2^+/−^ mice, double Robo1/2^+/−^ knockout; HGC, human granulosa cells; FSHR, follicle-stimulating hormone receptor. Scale bars = 100 μm in (A) and 50 μm in (E–F).

To further confirm these observations, we measured apoptosis in primary cultures of mouse and human granulosa cells (Fig. 6). We first performed the primary culture of the mouse granulosa cells that were isolated from the wild-type and Robo1/2^+/−^ knockout ovaries, and we found somemorphological differences between the wild-type and Robo1/2^+/−^ knockout granulosa cells, the Robo1/2^+/−^ knockout granulosa cells are more vigorously growth (Fig. 6A). And, the number of granulosa cells in the Robo1/2^+/−^ knockout group was increased compared to the wild-type ovaries (Fig. 6B). Flow cytometry data showed that the reduced number of granulosa cells in the primary culture wasa result of the decline incell apoptosis as indicated by red squares in Fig. 6C and 6D. We next used primary cultures of human granulosacells to confirm these observations (Fig. 6E, HE staining). The positive FSH receptor response in the IHCexperiments confirmed that these weregranulosa cells because the FSH receptor is a marker for ovarian granulosa cells (Fig. 6F). Robo1 and Robo2 were down-regulated by siRNA and were not altered in the negative control using mock siRNA (Fig. 6G), and this down-regulation was associated with reduced cell apoptosis compared to wild-type (Fig. 6H–I). Thus, both *in vivo* and *in vitro* experiments indicated that cell apoptosis was reduced following the partial knockdown of Robo1/2 expression in ovarian granulosa cells.

### Robo1/2^+/−^ knockout increased ovarian angiogenesis

**Figure 7:**
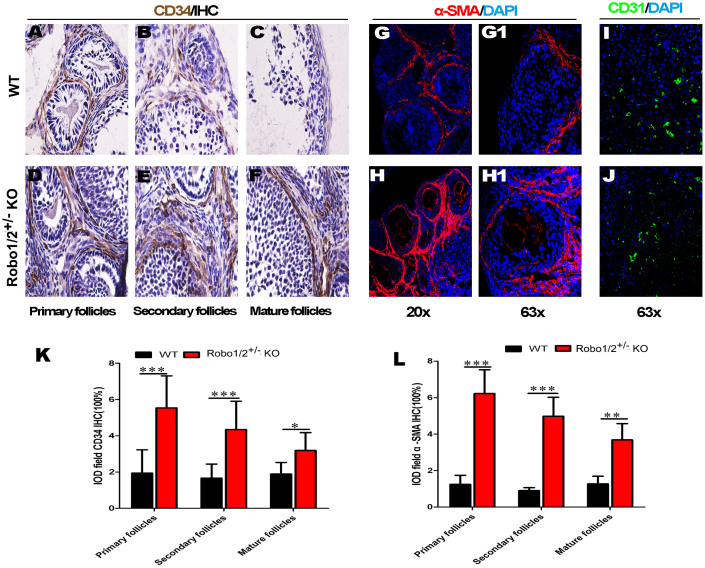
The increase in angiogenesis in ovaries of the Robo1/2^+/−^ knockout mice. (A–C): The immunocytochemistry against CD34 was performed on the vertical sections of the wild-type mouse ovary. The photographs were taken at the site of primary (A), secondary (B), and mature (C) follicles. (D–F): The immunochemistry against CD34 was performed on the vertical sections of the Robo1/2^+/−^ knockout mouse ovary. The photographs were taken at the site of primary (D), secondary (E), and mature (F) follicles. (G): The bar chart showing the comparison of integral optical density (IOD) for CD34 expression in primary (WT n = 10, Robo1/2 n = 7), secondary (WT n = 11, Robo1/2 n = 1), and mature follicles (WT n = 5, Robo1/2 n = 5). (H–I): The fluorescent immunostaining against SMA was performed on the vertical sections of the wild-type (H) and Robo1/2^+/−^ knockout (I) mouse ovaries, followed by a DAPI counterstain. (H1–I1): The high-magnification images from the sites indicated by dotted squares in (G) and (H), respectively. (J): The bar chart showing the comparison of IOD for SMA expression in the primary (WT n = 5, Robo1/2 n = 5), secondary (WT n = 3, Robo1/2 n = 5), and mature (WT n = 4, Robo1/2 n = 4) follicles. (K–L): The fluorescent immunostaining against CD31 was performed on the vertical sections of the wild-type (K) and Robo1/2^+/−^ knockout (L) mouse ovaries followed by a DAPI counterstain. Abbreviations: IOD, integral optical density; WT, wild-type; ROBO1/2^+/−^ mice, double Robo1/2^+/−^ knockout. Scale bars = 20 μm in (A–F), 200 μm in (H–I), 50 μm in (H1–I1), and 50 μm in (K–L).

It has been well-established that apoptosis is involved in the biological process of follicular atresia in which the majority of follicles are eliminated while some follicles are elected as dominant follicles. This process is strictly regulated by FSH through the suppression of granulosa cell apoptosis^19^. Therefore, a reasonable hypothesis is that the level of FSH in ovarian follicles should be related to angiogenesis in the ovaries, and well-known that Robo/Slit signaling are related to vascular. The CD34 antigen is present in immature hematopoietic precursor cells, so we examined its expression in ovarian tissue. We found that Robo1/2^+/−^ knockout (Fig. 7D–F) significantly increased CD34 expression in the primary, secondary, and mature follicles compared with wild-type (Fig. 7A–C). Pericytes are known to stabilize blood vessels and the α-SMA antigen is present in pericytes, we used α-SMA to further assess the effects of Robo1/2^+/−^ knockout on angiogenesis. The same localization images of the ovaries showed that α-SMA-positive cells in theRobo1/2^+/−^ knockout (Fig. 7H–H1) were substantially increased compared with wild-type (Fig. 7G–G1). The increase in angiogenesis occurred in the primary, secondary, and mature follicles (not all data shown). The immunofluorescent staining for CD31, which is normally expressed on endothelial cells, alsoindicated more small blood vessels in theRobo1/2^+/−^ knockout ovary (Fig. 7J compared with the wild-type ovary (Fig. 7I. The IHCdata suggest that ovarian angiogenesis was promoted by the Robo1/2^+/−^ knockout in the mouse ovary.

## Discussion

Slit/Robo signaling exerts its effects during tissue morphogenesis. Thus, the disruption of certain Slit and/or Robo proteins is often associated with tumor formation in different tissues. Dickinson et al. demonstrated that Slit/Robo signaling could also performcrucial functions in the reproductive system^13^. The expression of Slit/Robo in the pre-granulosa cells and the oocytes of the developing primordial follicle indicate that Slit/Robo signaling might function through both autocrine and paracrine interactions^14^. To further explore the role of Slit/Robo in reproductive biology, we generated a partial knockout of the Robo1/2 genes in mice because full knockout was embryonically lethal. We found that the partial lack of Robo1/2 resulted ina greater number of offspring compared with the wild-type mice (Fig. 1C–D). Multiple factors are involved in reproductive capacity, and gamete maturation in the gonads ismost likelythe initial event affecting reproductive ability. Weyers et al. demonstrated that Slit/Robo signaling ispivotal for proper gonad formation, although gonad formation has been found to be regulated by multiple and independent pathways^20^. Thus, we have examined *in vitro* gamete vitality by measuring the rates of ovum maturation and fertilization and the rates of zygote cleavage and degradation in the 4-week-old (Supplementary Fig. 2A–D) and10-week-old (Supplementary Fig. 2E–H) wild-type and Robo1/2^+/−^ knockout mice. The results indicated that there were no significant alterations in gamete viability following the partial knockout of the Robo1/2 genes. Furthermore, Cy treatment normally leads to ovarian follicle exhaustion^21^, but the Robo1/2^+/−^ knockout could prevent this (Supplementary Fig. 1A), and this suggests that normal Slit/Robo signaling might promote follicle atresia via effects on granulosa cell apoptosis (Supplementary Fig. 1C). This finding also verified the hypothesis that Slit/Robo signaling influences fertility.

In the subsequent analysis of follicle development, we determined that the increase in offspring number might be closely related to the development of activated follicles (Fig. 3). Thus, Robo1/2^+/−^ knockout increased follicle maturity and decreased follicle atresia (Fig. 3E), and this indicated that more offspring were born to mice with a partial lack ofRobo1/2 genes. In general, this observation is consistent with previous reports in which over-expression of Slit/Robo in the ovary resulted in a distinct reduction in the number of proliferating oocytes^14^. In this study, the Robo1/2 genes were partially knocked out in mice, but in wild-type mice both the Robo1 and Robo2 genes were strongly expressed in the ovarian granulosa cells (Fig. 4A–B), and their expression tended to increase as the follicles matured (Fig. 4C–J). This finding suggests that Slit/Robo signaling is intimately correlated with follicle maturation and atresia because granulosa cells are indispensable for the normal development of the ovarian follicles.

It is well known that only a few follicles from the entire follicle pool are able to mature and eventually ovulate in each estrous cycle, i.e., the vast majority of follicles undergo atresia rather than developing further following the formation of an antrum. Only the selected follicles become ovulatory follicles, and ovulation is then followed by transformationinto the corpora lutea. It is during the process of follicular atresia that granulosaand lutein cells undergo apoptosis^22^,^23^,^24^. This process was the impetus forus to assess apoptosis in theovaries ofwild-type and Robo1/2^+/−^ knockout mice (Fig. 5). The TUNELassay demonstrated that apoptosis in the granulosa cells from the Robo1/2^+/−^ knockout follicles was substantially reduced compared with those fromwild-type follicles (Fig. 5A–B), and apoptosis was reduced in all stages of the developing follicles (Fig. 5C). The reduction in apoptosis induced by Robo1/2^+/−^ knockout was verified by IHC against caspase-3 (Fig. 5D–E), which is another marker for apoptosis. This is because granulosa cells, which comprise the layer of small cells that form the wall of the ovarian follicle, are fundamental in determining the follicle's fate. At the same time, the influence of Robo on apoptosis is again the important factor that supports our hypothesis in this study. Therefore, we double-checked the effects of Robo1/2 knockdown *in vitro* mouse and human granulosa cell cultures (Fig. 6). The *in vitro* experimental assay demonstrated that Robo1/2^+/−^ knockout reduced the apoptosis-positive cell population (Fig. 6C), and this was also reflected in the greater number of cells in the Robo1/2^+/−^ knockout mouse granulosa cell culture (Fig. 6B). In the human granulosa cell culture, there were fewer apoptotic cells when Robo1/2 was partially knocked out using Robo1/2-siRNAcompared with the cells receiving mock siRNA. Thus the experimental results from the *in vivo* and *in vitro*Robo1/2^+/−^ knockout were the same, and we conclude that partial lack of Robo1/2 leads to are duction of granulosa cell apoptosis.

Riaz et al. reported that the somatostatin receptor 2 sub-type regulates granulosacell apoptosis and proliferation through selective constitutive action that is independent of somatostatin^25^. Wnt signaling has also been demonstrated to negatively regulate follicular development via components of the Foxo3a signaling pathway^26^. The proliferation and aromatization capacity of rat granulosacells are stimulated by both FSH and TGF-beta^27^. FSH has been demonstrated to regulate granulosacell proliferation through its influence on micro-RNA expression. From previously published studies, we can reasonably speculate that the determination of follicle fate (to undergo either maturation or atresia) relieson the levels of FSH or other hormones. Thus, the levels of FSH or other hormones delivered by ovarian angiogenesis most likely play a crucial role in the determination of follicle fates. Thus, we can evaluate the hormone level, such as FSH, through the assessment of the local angiogenesis in ovaries if we cannot directly measure FSH levels in the follicles. In addition, the interaction between the secreted Slit ligand and Robo receptorhas been implicated in the regulation of cell death and angiogenesis^13^,^28^. It is not surprising, therefore, that ovarian angiogenesis was detected by IHC against CD34 and IF staining against SMA and CD31 (Fig. 7). The angiogenesis data demonstrated that Robo1/2^+/−^ knockout could lead to anincrease of angiogenesis around developing follicles (Fig. 7G, J). These findings imply that our hypothesis regarding the hormone level alterations induced by Robo1/2^+/−^ knockout has amorphological basis in angiogenesis. Further investigation will be required to explore the mechanism for how Robo1/2^+/−^ knockout can affect angiogenesis in ovaries. And another question is whether or not higher level of progesterone in Robo1/2^+/−^ knockout is associated with less follicle apoptosis.

**Figure 8:**
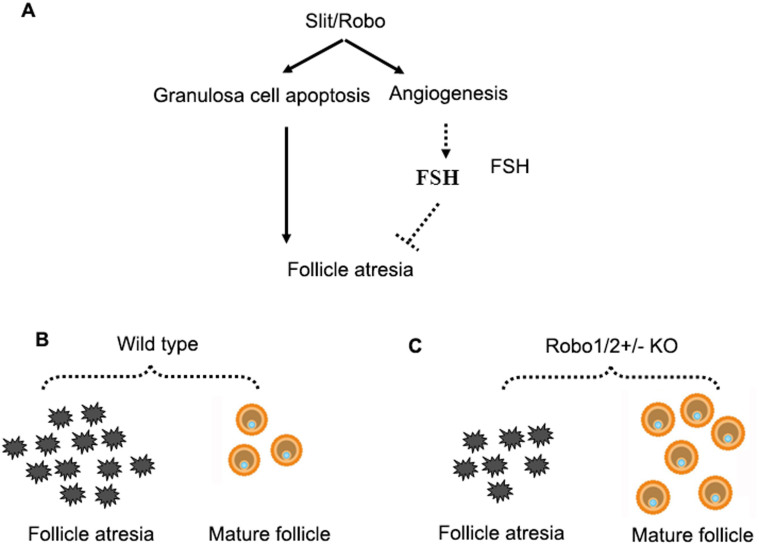
A proposed model of the potential mechanisms of Slit/Robo signaling in the regulation of ovarian follicle atresia. (A): The Robo1/2 knock-out would decrease the granulosa cell apoptosis and angiogenesis, which lead to follicles atresia. Figure B and C be used to show Robo1/2 knockout results in more number of mature follicles.

In summary, we have used a transgenic mouse model to demonstrate the potential role of the Slit/Robo signaling pathway in the reproductive capacity in mice. The hypothesis is illustrated in Fig. 8. The Slit/Robo pathway might be involved in the regulation of ovarian follicle development and atresia by targeting the granulosa cells for apoptosis. Another potential pathway is most likelythe effect on ovarian angiogenesis, which alters FSH and other hormone levels. These hormone levels, in turn, modulate granulosa cell proliferation and apoptosis. Therefore, upon induction ofRobo1/2^+/−^ knockout, more mature follicles form and less follicular atresia occurs and this leads toa greater number of offspring being born to mice with a partial lack of Robo1/2. Further molecular biological experiments are needed to better understand the role of the Slit/Robo pathway in reproductive biology.

## Additional information

**How to cite this article**: Li, J. *et al.* Robo1/2 regulate follicle atresia through manipulating granulosa cell apoptosis in mice. *Sci. Rep.* 5, 9720; DOI:10.1038/srep09720 (2015).

## Supplementary Material

Supplementary Information dataset 1
