# Supplementary material for: Robo1/2 regulate follicle atresia through manipulating granulosa cell apoptosis in mice
Source: Sci Rep. 2015 May 19;5:9720. doi: 10.1038/srep09720 (PMC4437031; doi:10.1038/srep09720)
Supplement: Supplementary Information — dataset 1 [file srep09720-s1.doc]

**Robo1/2 regulate follicle atresia through manipulating granulosa cell apoptosis in mice**

*Jiangchao Li^1#^, Yuxiang Ye^1#^, Renli Zhang^3#^, lili Zhang^3,4^, Xiwen Hu^1^, Dong Han^3^，Jiayuan Chen^1^, Xiaodong He^1^, Guang Wang^2^, Xuesong Yang^2*^, Lijing Wang^1*^*

***Supporting Information***

***
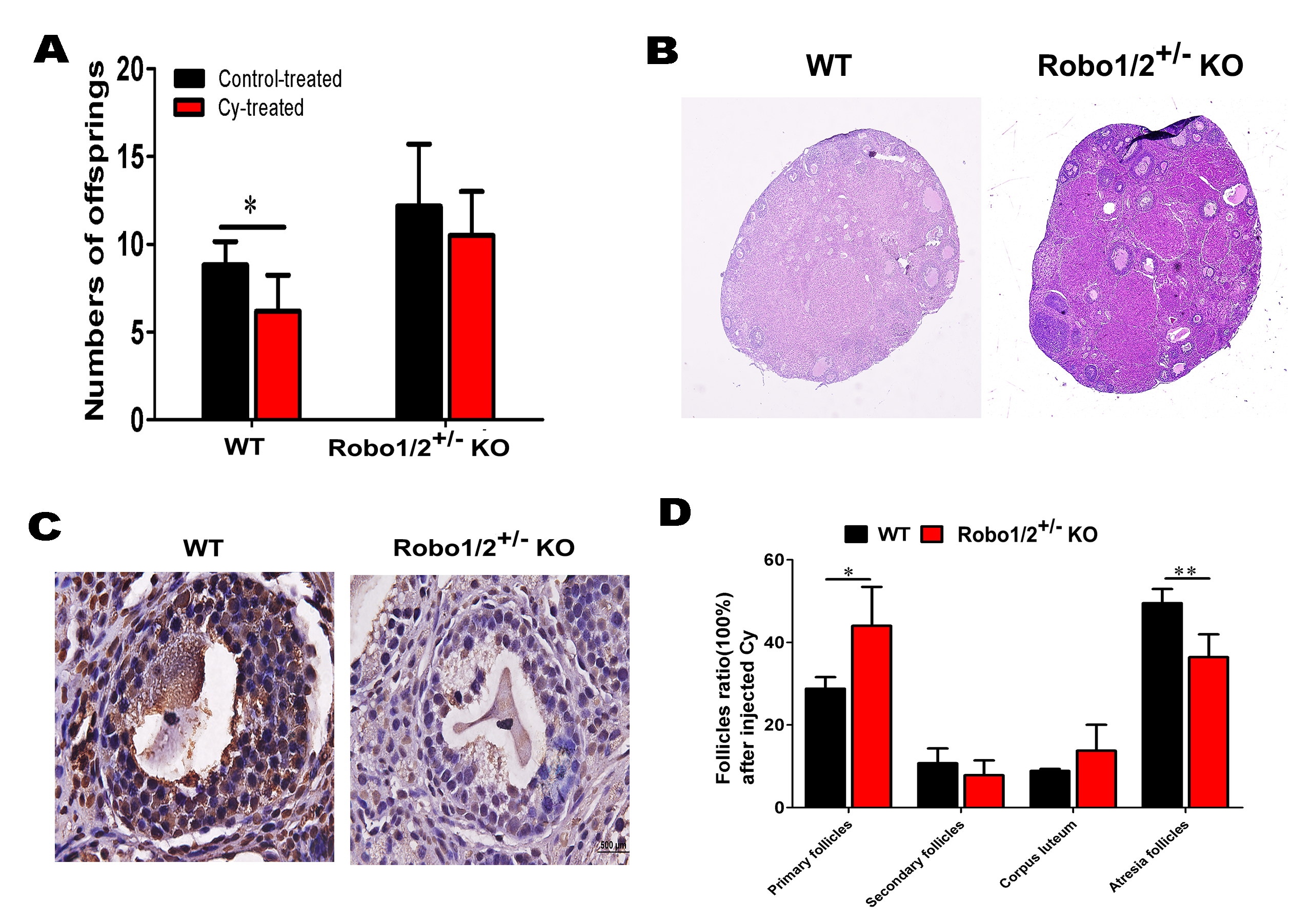
***

***Supplementary Figure 1: The protective role of Slit/Robo signaling on mouse fertility reduction induced by Cyclophosphamide treatment.***

**A**: The bar chart showing the mouse offspring number from the wide-type (control treated n=6, Cy treated n=5) and Robo1/2^+/-^ knockout mice (control treated n=6, Cy treated n=5) mice following the injection of Cy. **B**: The H&E staining was performed on the vertical sections of the wide-type and Robo1/2^+/-^ knockout mouse ovaries. **C**: The TUNEL assay was performed on the vertical sections of the wide-type and Robo1/2^+/-^ knockout mice mouse ovaries following the treatment of Cy. The images focused on the ovarian follicles. **D**: The bar chart showing the ovarian follicle number from the wide-type (n=4) and Robo1/2^+/-^ knockout (n=4) mice following the injection of Cy. Abbreviations: WT, wide-type; Robo1/2^+/-^ knockout, double partial Robo1/2 knock-out; WT-Cy, wide-type + Cy; DKO-Cy, Robo1/2^+/-^ knockout + Cyclophosphamide. Scale bars = 200 μm in B and 20 μm in C.

**
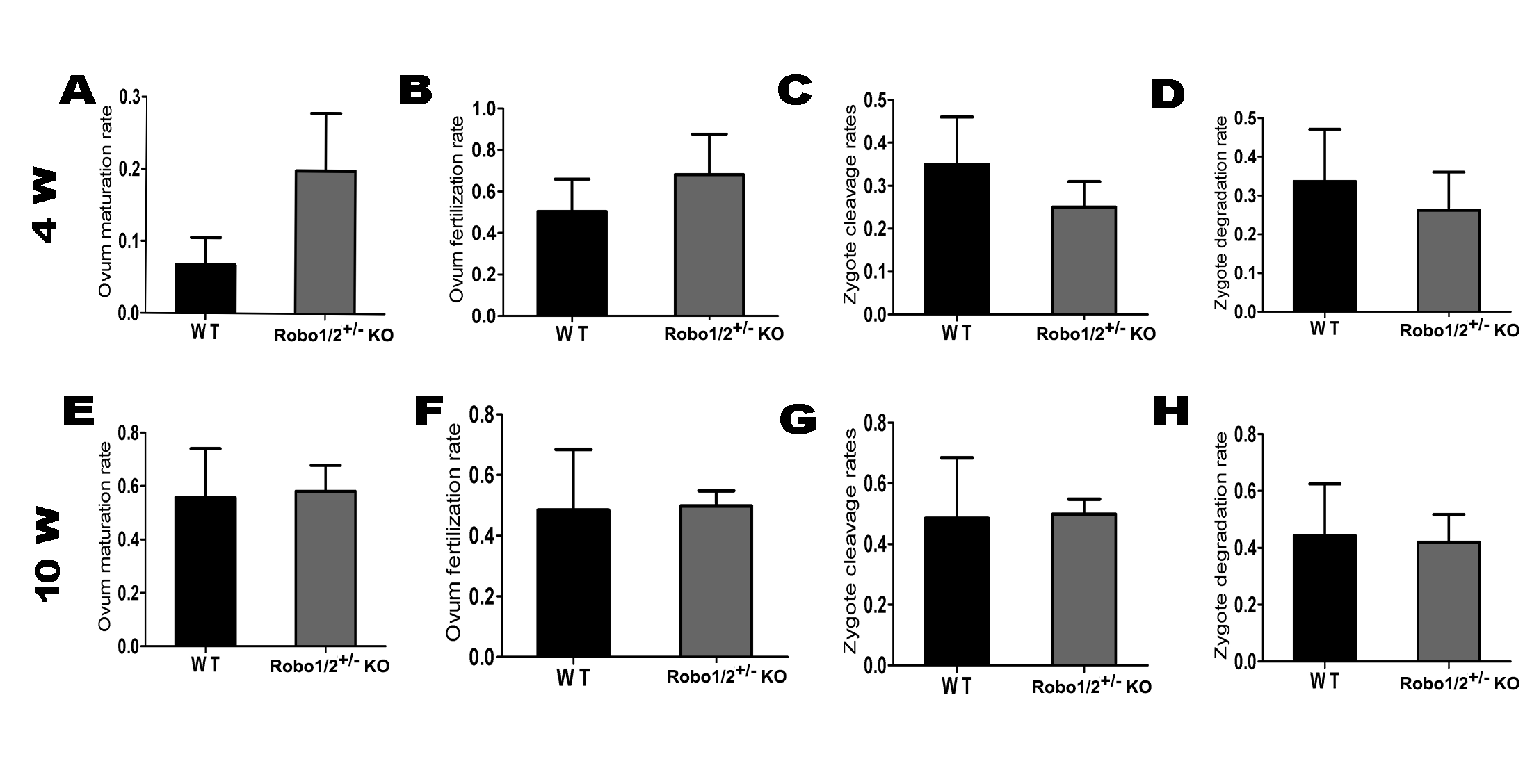
**

***Supplementary Figure 2: The fertility determination from the wide-type and Robo1/2^+/-^ knockout mice.***

**A-D**: The ovum maturation rate (A, WT n=8, Robo1/2 n=9), ovum fertilization rate (B, WT n=8, Robo1/2^+/-^ KO n=9), zygote cleavage rate (C, WT n=8, Robo1/2^+/-^ KO n=9) and zygote degradation rate (D, WT n=8, Robo1/2^+/-^ KO n=9) were detected in the 4-week wide-type and partial Robo1/2^+/-^ KO mice. **E-H**: The ovum maturation rate (E, WT n=6, Robo1/2^+/-^ KO n=3), ovum fertilization rate (F, WT n=6, Robo1/2^+/-^ KO n=3), zygote cleavage rate (G, WT n=6, Robo1/2^+/-^ KO n=3) and zygote degradation rate (H, WT n=6, Robo1/2^+/-^ KO n=3) were detected in the 10-week wide type and Robo1/2^+/-^ KO mice.
